# Oxidative DNA double strand breaks and autophagy in the antitumor effect of sterically hindered platinum(II) complexes in NSCLCs

**DOI:** 10.18632/oncotarget.15944

**Published:** 2017-03-06

**Authors:** Feihong Chen, Xinyi Wang, Xiufeng Jin, Jian Zhao, Shaohua Gou

**Affiliations:** ^1^ Pharmaceutical Research Center and School of Chemistry and Chemical Engineering, Jiangsu Province Hi-Tech Key Laboratory for Biomedical Research, Southeast University, Nanjing 211189, China

**Keywords:** platinum(II) complexes, N^1^,N^2^-diisobutyl moiety, ROS, double strand breaks, MDC1/aprataxin

## Abstract

A series of novel platinum(II) complexes with (1R,2R)-N^1^,N^2^-diisobutyl-1,2-diaminocyclohexane as a carrier ligand, while N^1^,N^2^-diisobutyl moiety serving as steric hindrance were designed, synthesized and characterized. The *in vitro* biological assays demonstrated that complex 3 had increased cytotoxicity against lung cancer cells, especially non-small-cell lung cancer (NSCLC) compared to its mono-substituted complex 3a, indicating that the sterically hindered alkyl moieties have significant influences on its antitumor property. However, the mechanism still remains unclear. The further studies revealed that complex 3 could induce ROS overproduction, severe DNA double strands breaks and inhibit the activation of DNA damage repair proteins within nucleus, leading to cell-cycle arrest and cell death. Moreover, complex 3 could induce autophagy *via* the accumulation of autophagic vacuoles and alterations of autophagic protein expression. Interestingly, the ROS scavengers, N-acetyl-cysteine (NAC) could reverse complex 3-induced DNA double strands breaks and autophagic responses more significantly compared to complex 3a. The results demonstrated that the ROS generation plays an important role in the DNA double strands breaks and autophagic responses in the antitumor effect of complex 3 with N^1^,N^2^-diisobutyl moiety. Our study offered a novel therapeutic strategy and put new insights into the anticancer research of the complexes with N^1^,N^2^-diisobutyl moiety served as steric hindrance.

## INTRODUCTION

In the past three decades, lung cancer was the most frequently diagnosed cancer and the leading cause of cancer death among males as well as females in the developed countries [1]. The mortality rate for lung cancer is 610.2 per 100,000 in China, which accounts for more than one-fifth of all cancer deaths [2]. Non-small-cell lung cancer (NSCLC) occupied approximately 85% of all these lung malignancies [3]. Standard first-line chemotherapeutic scheme in NSCLC patients was platinum-based combination chemotherapy, which means that platinum compounds were combined with the third generation chemotherapy agents such as gemcitabine, vinorelbine, camptothecin [5–7]. However, side effects of the treatments must be considered carefully. Platinum compounds, for example, are chartered with considerable gastrointestinal toxicities including nausea, vomiting and delayed diarrhea [8]. Moreover, concern with chemotherapy agents is arisen for their association with neurotoxicity, nephrotoxicity and gastrointestinal toxicity [8–11]. Disappointingly, when these targeted agents were combined with chemotherapy agents, neither overall survival nor clinical response rate in phase III clinical trials was significantly developed, while the increased risk of the treatment instead [8, 12, 13]. Thus, it is urgent to find novel drugs with high efficiency rate and low organ toxicity.

**Scheme 1:**
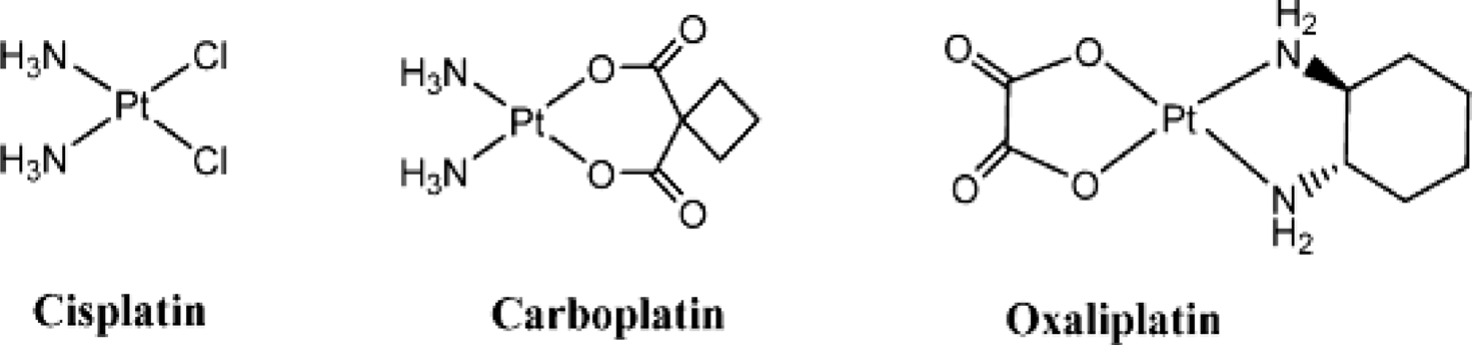
Structures of cisplatin, carboplatin, oxaliplatin

Platinum(II)-based drugs, such as cisplatin, carboplatin and oxaliplatin (Scheme 1) are potent cytotoxic agents as the first line treatment for a variety of malignancies despite of severe side effects and innate or acquired resistance. With the success of oxaliplatin, a number of 1,2-DACH derivatives have been designed and tested for their anticancer activities [12–18]. Among them, a small number of symmetric N^1^,N^2^-disubstituted 1,2-DACH derivatives have been used as carrier ligands [13, 14, 19, 20]. Our group has previously designed a series of platinum-based complexes bearing N-mono-substituted 1*R*,2*R*-diaminocyclohexane derivatives as carrier ligands which showed optimistic antitumor activity compared with oxaliplatin [21]. Moreover, we synthesized several Pt(II) complexes of (1*R*,2*R*)-N^1^,N^2^-dibutyl-1,2-diaminocyclohexane with two n-butyl branches as steric hindrance, and the *in vitro* cytotoxicity detections indicated the complexes showed potent antitumor activity [22]. Thus we believed that the increase of sterically hindered effect of the platinum complexes could improve the cytotoxic activity and decrease the side effects, then we intend to introduce two alkyl moieties to the 1*R*,2*R*-DACH skeleton to further study the sterically hindered effect of the platinum complexes.

Autophagy is an evolutionarily conserved process for degrading long-lived and misfolded proteins, damaged and dysfunctional organelles, and foreign particles [23]. During autophagy, double-membrane vacuoles, called autophagosomes, are formed in the cytoplasm. Microtubule-associated protein light chain 3 (LC3) is essential for the formation of autophagosomes and is critical for the progression of autophagy. Therefore, LC3 is a consistent marker of autophagy [24]. Cytoplasmic components embedded in autophagosomes are delivered and degraded in an autolysosome structure [25]. Autophagy is critical for the maintenance of homeostasis and in certain diseases, such as viral infections, neurodegeneration, cardiovascular diseases, cancers and aging [26–28].

Oxidative stress resulted from excessive generation of ROS induces multiple biological responses, such as DNA damage inclusive of DNA double-strand breaks (DSBs) and single-strand breaks (SSBs), cell cycle arrest, autophagy and apoptosis. DNA damage response activated ATM, Chk1/2, p53 and subsequently resulted in cell cycle arrest and apoptosis. As key mediators of DSBs and SSBs repair, MDC1/aprataxin and XRCC1 could be activated in nucleus [29]. Moreover, previous studies demonstrated that the proliferation inhibition of cancer cells could be mediated by autophagy via ROS generation. Thus, the level of ROS has been widely elevated by cellular cytotoxic agent that was demonstrated to trigger autophapic cell death. To determine whether Pt(II) complexes of (1R,2R)-N^1^,N^2^-dibutyl-1,2-diaminocyclohexane could trigger ROS-mediated DNA damage and autophagic responses, we detected the level of ROS generation and DNA damage, the autophagic vacuoles and protein expression *in vitro* and *vivo*. Recently, the synthesized Pt(II) complexes of (1*R*,2*R*)-N^1^,N^2^-dibutyl-1,2-diaminocyclohexane with two isobutyl branches as steric hindrance have been evaluated for their *in vitro* cytotoxicities against human tumor cell lines. The flow cytometric detection was carried out to test the apoptotic effect and cell cycle arrest, ROS level of the complexes in the selected cancer cell lines along with the disfunction of mitochondria, while the comet and western blot assays determined the DNA damage level and the autophagic responses. Generally, the study summarizes the influence of the resulting platinum complexes on ROS-meditated DNA damage and autophagy, with the induction of cell apoptosis and death.

## RESULTS

### Synthesis and characterization

**Scheme 2:**
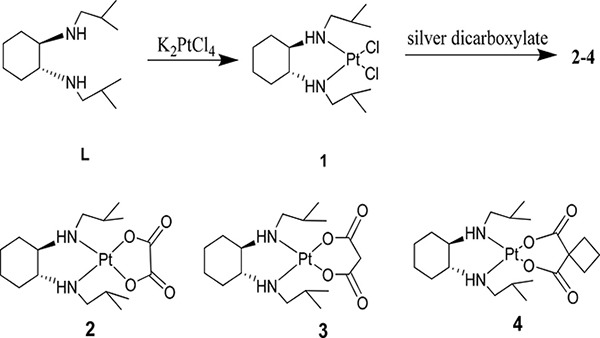
Synthetic pathway to target complexes 2-4

The four platinum(II) complexes were prepared following the procedures listed in Scheme 2. Under the protection of nitrogen and dark, an aqueous solution of K_2_PtCl_4_ was added to L to generate complex 1. The completion of the reaction took a long time than expected, indicating that alkyl species have caused hindrance for the ligand to bind the metal atom. Further reactions of complex 1 with the corresponding silver dicarboxylate were carried out in water to form (1*R*,2R)-N^1^,N^2^-diisobutyl-1,2-DACH-Pt(II) conjugates (complexes 2–4).

The newly synthesized platinum(II) complexes were characterized by elemental analysis, IR, ^1^H NMR spectra and electrospray ionization mass (ESI-MS) spectroscopy. In the infrared spectra, the N-H stretching vibrations of complexes 1–4 were obviously shifted to lower frequencies as compared with the free ligand, due to the coordination of the amino group with Pt(II) ions. Besides, the C = O vibration of complexes 2–4 appeared in the range from 1579 to 1646 cm^−1^, which were characteristic of coordinated dicarboxylates, while the C-O feature appeared in the range of 1350–1396 cm^−1^. All the ^1^H NMR spectral data are reasonably attributed to the molecular structures of the synthesized compounds. Furthermore, all the platinum(II) complexes showed 100% of [M+Na]^+^ or [M+Cl]^−^ peaks in ESI-MS spectroscopy.

### *In vitro* cytotoxic activity

The cytotoxicity of the synthesized complexes was evaluated via MTT assays toward HepG2, SGC-7901, A549, HCT-116 cancer cell lines and L02 normal liver cell line with oxaliplatin as a positive control. The corresponding IC_50_ values are presented in Table 1. As shown in Table 1, it is clear to find that complex 1 had considerable cytotoxicity against the tested cell lines, except A549. Complex 2 showed selective activity against certain tested cell lines (A549, HCT-116) while complex 4 has nearly no antitumor activity against all cancer cell lines. Interestingly, complex 3 showed better cytotoxicity activity against all the tested cell lines, especially against A549 compared to oxaliplatin and its mono-substituted complex 3a, [(1*R*,2*R*)-N^1^-(2-methylpropyl)-1,2-cyclohexanediamine-N,N’](malonato-O,O’) (Table 2), while complex 3 showed lower cytotoxicity against L02 cell line with the IC_50_ of 82.57 ± 3.95 μM compared with the positive control oxaliplatin of 42.9 ± 3.54 μM. By comparing the cytotoxicity of complexes 1–4 to oxaliplatin, we can conclude that the existence of alkyl moieties, on each amino group of 1,2-DACH has a significant influence on the antitumor property of complex 3 up to 7.94-folds and 5.37-folds increased in contrast to oxaliplatin and complex 3a. The results reveal that the steric hindrance plays a significant role in the cytotoxicity of complex 3.

**Table 1:** *In vitro* cytotoxicity of complexes 1-4 and oxaliplatin

Complexes	IC_50_ (μM)^a^					
	HepG2	SGC-7901	HCT-116	A549	NCI-H1299	L02
Complex 1	34.07 ± 2.55	44.60 ± 3.24	70.55 ± 5.56	> 100	> 100	147.75 ± 10.6
Complex 2	> 100	> 100	37.03 ± 2.76	28.38± 1.45	> 100	> 100
Complex 3	52.90 ± 4.37	38.63 ± 2.49	38.60 ± 2.67	14.17 ± 1.22	20.92 ± 1.36	82.57 ± 3.95
Complex 4	> 100	> 100	> 100	> 100	> 100	> 100
Oxaliplatin	16.6 ± 0.56	24.2 ± 0.35	19.1 ± 1.89	112.5 ± 11.3	62.4 ± 2.35	42.9 ± 3.54

*^a^*Mean value ± standard deviation from three independent experiments; IC_50_ defined after 48 h drug exposure.

**Table 2:** *In vitro* cytotoxicity of complex 3 and 3a exposed for 48 h

Complexes	IC_50_ (μM)				
	HepG2	MCF-7	HCT-116	A549	NCI-H1299
3	52.90 ± 4.37	32.50±2.9	38.60 ± 2.67	14.17 ± 1.22	20.92 ± 1.36
3a^a^	> 100	> 100	65.8 ± 4.95	77.5 ± 5.04	94.85 ± 6.74
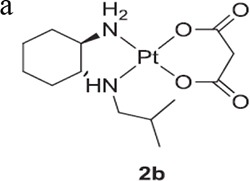					

### Complex 3 improved the cellular uptake of Pt in NSCLCs

**Figure 1:**
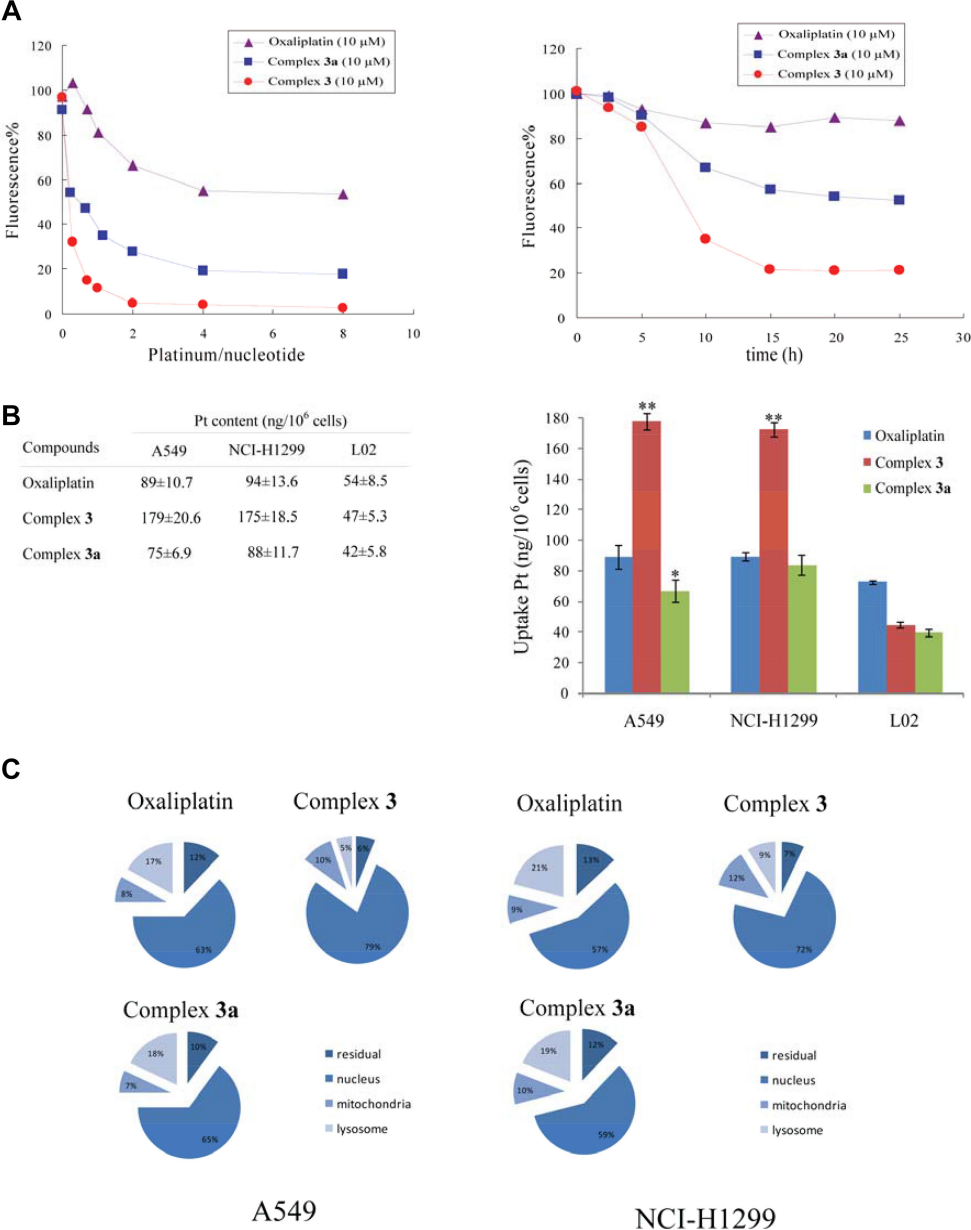
Reactions of complex 3 with DNA by reduction (**A**) The different Pt/nucleotide ratios and time of DNA platination. All the reactions were conducted with 0.01 mg/mL DNA in 10 mM NaClO_4_ in 10 mM phosphate buffer (pH = 7.4) at 37°C for 24 h and then add 0.04 mg/mL EtBr before the fluorescence measurements with the excitation wavelength of 546 nm and the emission wavelength of 590 nm. (**B**) The cellular uptake of oxaliplatin, complex 3, complex in A549, NCI-H1299 and L02 cells. (**C**) The accumulation of the measured compounds in mitochondria, lysosomes and nucleus of A549 and NCI-H1299 cells. The levels of Pt in cancer cells were detected by ICP-MS after 4 h incubation with the treatments of evaluated complexes at 10 μM. Results are representative of at least three independent experiments and shown as the mean ± S.D. *P < 0.05, **P < 0.01 compared with oxaliplatin-treated groups.

The better cytotoxicity of complex 3 owing to the DNA platination could be detected in the presence of EtBr as a probe via fluorescent measurements, because their DNA platination could prevent the formation of EtBr-DNA complex, resulting in its stoichiometric loss of fluorescence. It was observed in Figure 1A that complex 3 could cause more fluorescent intensity change compared to complex 3a, while oxaliplatin could definitely lead to a little loss of fluorescent intensity under the same condition. The above results indicated that complex 3 can induce DNA platination to exert its cytotoxic effect.

One of the main factors to determine the mechanism of cytotoxic activity of a metal-based drug is its ability to cross the cell membrane and to accumulate in cancer cells. Therefore, with the aim of correlating cellular accumulation, we investigated the cellular uptake of the platinum complexes in A549, NCI-H1299 cancer cells and L02 normal cell. After 8 h treatment of oxaliplatin, complex 3 and complex 3a of 10 μM, the platinum contents in these cancer cells and normal cell were analyzed via ICP technique. As Figure 1B shown, the Pt accumulations of complex 3 in A549 and NCI-H1299 cancer cells were increased compared to complex 3a (2.4 and 2.0 times, respectively), and higher than that of oxaliplatin (2.0 and 1.9 times, respectively), while there is 1.1-folds lower in HUVEC normal cells compared to oxaliplatin. Moreover, the amount of Pt in the different subcellular compartments of cancer cells was quantified to fetch a more detailed picture of Pt-based complexes intracellular localization (Figure 1C). As expected for oxaliplatin, which is known to induce cell death by forming adducts on nucleus DNA, 63% of the total intracellular Pt was located in the nucleus of A549 cancer cells, while 57% in NCI-H1299 cancer cells. In contrast, the ratio was 79% and 72% for complex 3 in A549 and NCI-H1299 cells, respectively, while there is 1.2-folds higher in both cancer cells compared to its mono-substituted complex 3a. Thus, these results indicated that the increased cellular uptake of complex 3 compared to its mono-substituted complex 3a attributed to its unique structure of steric hindrance.

### Complex 3 inhibited cells viability and induced cell apoptosis in NSCLC cells

**Figure 2:**
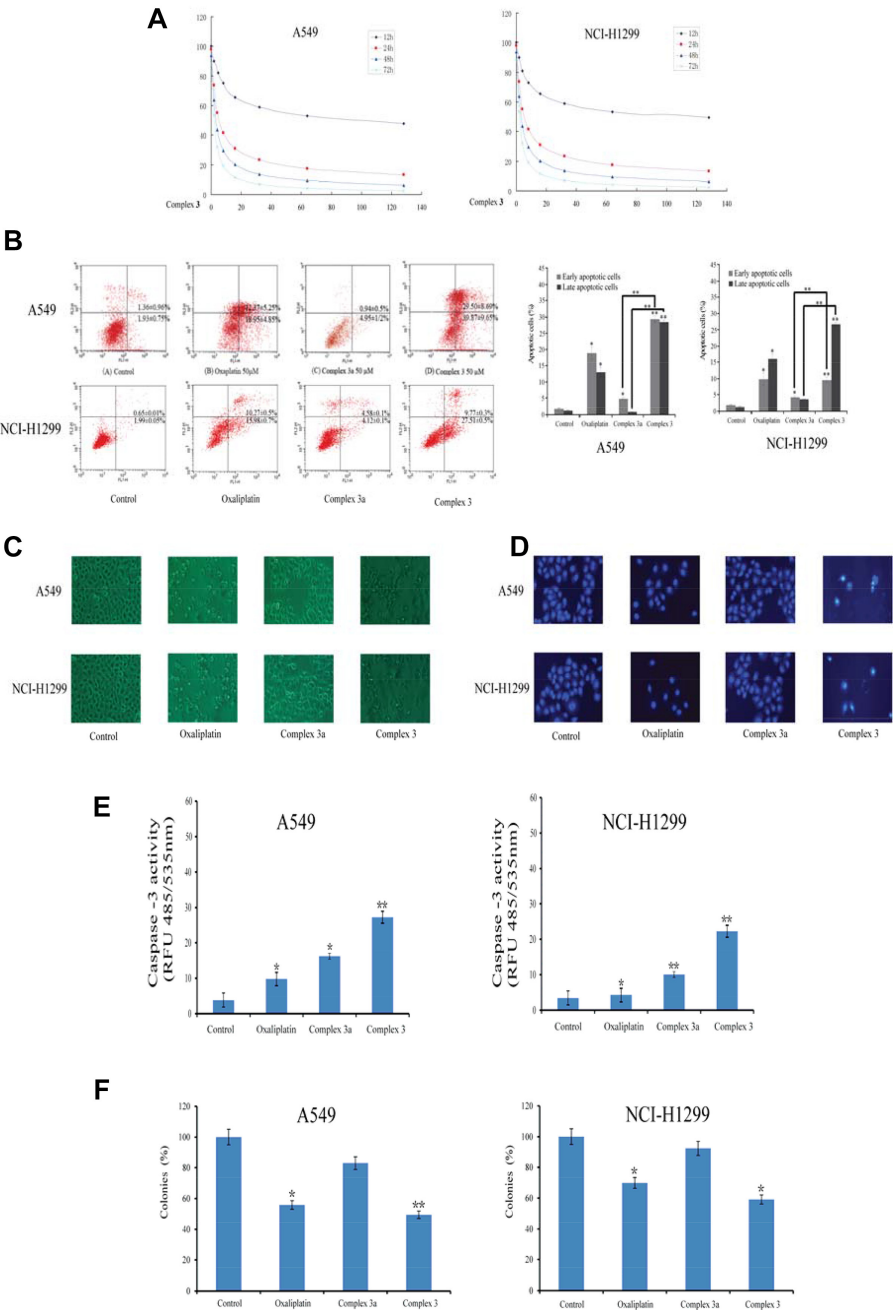
Cellular responses of oxaliplatin, complex 3 and complex 3a in A549 and NCI-H1299 cancer cells All the complexes were used at the fixed concentration of 10 μM for 24 h. (**A**) The growth inhibition effect of complex 3 on A549 and NCI-H1299 cancer cells on 12, 24, 48 and 72 h. (**B**) Apoptosis inducing property of the measured complexes by Annexin V-FITC/PI staining of cancer cells. The Y-axis shows the PI-labeled population and the X-axis shows FITC-labeled Annexin V-positive cells. (**C**) Morphological changes in A549 and NCI-H1299 cancer cells were observed under an inverted light microscope (original magnification 100 ×). (**D**) Apoptotic cells were observed by DAPI staining. (**E**) Analysis of caspase-3 activation in cancer cells following the treatment of measure compounds. (**F**) Long-term colony formation assays of A549 and NCI-H1299 cancer cells. Cells were grown in the presence of the measured complexes for 7 days. For each cell line, all dishes were fixed at the same time, stained and analyzed. Results are representative of at least three independent experiments and shown as the mean ± S.D. *P < 0.05, **P < 0.01 compared with control group.

The effects of complex **3** on the viability of NSCLC cancer cells including A549 and NCI-H1299 were detected respectively via MTT assays at time points of 12, 24, 48 and 72 h (Figure 2A). The results shown that complex **3** could inhibit the viability of the NSCLC cells. A549 and NCI-H1299 cells treated by complex **3** for 24 h within the concentrations ranging from 2 to 128 μM exhibit significant inhibition of cell viability in a concentration-dependent manner. The IC_50_ values for A549 and NCI-H1299 cells exposed for 24 h were 6.55 and 7.42 μM respectively, indicating that complex **3** could effectively suppressed the viability of these NSCLC cells for 24 h. Hence, the further studies were carried out on cancer cells exposed for 48 and 72 h.

Apoptosis is a crucial programmed death form of aging or damaged cells [30]. To confirm whether the cell viability-inhibition effects of complex 3 were resulted from apoptosis induction, the Annexin V/PI double staining assay was used on A549 and NCI-H1299 cells treated with oxaliplatin, complex 3a and complex 3 for 24 h. The results in Figure 2B implied that early and median apoptotic cells of A549 and NCI-H1299 cells were increased to 29.50%, 12.37%, 0.94% and 10.27%, 4.58%, 9.77% following treatment of oxaliplatin, complex 3a and complex 3 respectively with 50 μM for 24 h, while the late apoptotic cells were increased strikingly to 39.87%, 18.95%, 4.95% and 15.98%, 4.12%, 27.51%. Thus, the apoptotic rates of A549 and NCI-H1299 cancer cells treated with complex 3a and 3 indicated that the unique structure of the large steric hindrance of complex 3 performed higher apoptotic rates compared to its mono-substituted complex 3a, which was consistent with its cell viability inhibition due to the large steric hindrance of complex 3. The results of microscopic examination and DAPI staining (Figure 2C and 2D) revealed that the occurrence of cell shrinkage, cell rounding, chromatin condensation, and nuclei fragmentation after treatment with complex 3 of 10 μM. To further explore the impact of complex 3 on long-term clonogenic survival, colony formation assay was performed.

The caspase-3 activity in cancer cells was determined at concentration of 10 μM of oxaliplatin, complex 3a and complex 3 for 24 h. The cells incubated with complex 3 showed a high degree of caspase-3 activity compared to oxaliplatin in A549 and NCI-H1299 cancer cells (Figure 2E). The findings indicated that complex 3 might induce higher level of caspase-dependent apoptosis than oxaliplatin in NSCLC cancer cells. The results shown in Figure 2F revealed that complex 3 significantly reduced colony formation compared to its mono-substituted complex 3a in A549 and NCI-H1299 cancer cells, demonstrating that complex 3 could affects long-term clonogenic survival of NSCLC cells. Taken together, these experiments demonstrate that complex 3 could induce cell apoptosis in NSCLC cells and suppress long-term clonogenic survival.

### Complex 3 arrested cell cycle and decreased the mitochondrial membrane potential (ΔΨm) in NSCLC cells

**Figure 3:**
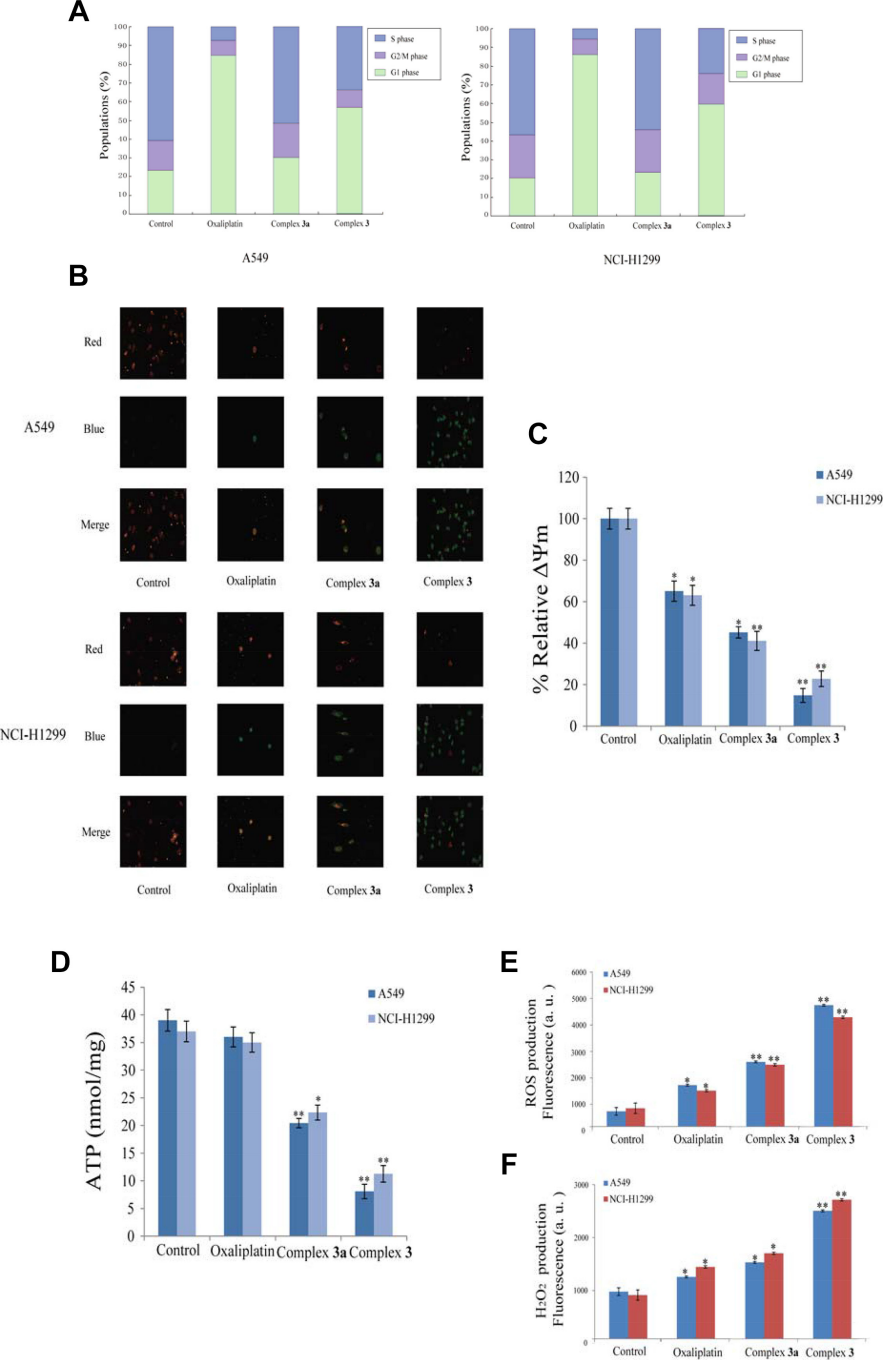
Complex 3 induces cell death and cell cycle changes in A549 and NCI-H1299 cancer cells (**A**) Cell cycle analysis upon exposure to complex 3. A549 and NCI-H1299 cancer cells exposed to the measured complexes for 72 h were stained with propidium iodide and subjected to flow cytometry analysis. The mitochondrial membrane potential (b, c) and the intracellular ATP (d) decreased in complex 3-treated A549 and NCI-H1299 cancer cells. (**B**) Cells were exposed to oxaliplatin, complex 3 and complex 3a (10 μM) for 24 h, stained with JC-1 and visualized under an inverted fluorescence microscope. Red fluorescence of JC-1 dimers was present in the cell areas with high MMP, while green fluorescence of JC-monomers was prevalent in the cell areas with low MMP. (**C**) Normalized JC-1 fluorescence change analyzed by flow cytometry. The median fluorescence intensity of each treatment group was normalized to the control group. (**D**) Cells were treated with oxaliplatin, complex 3 and complex 3a (10 μM), for 12 h and then the intracellular ATP was detected. (**E**) Intracellular ROS were measured by flow cytometry after 10 μM DCFH-DA staining. Geometric mean off luorescence intensity values were calculated and compared to that in DMSO controls. (**F**) Cells were exposed to 10 μM oxaliplatin, complex 3 and complex 3a, then H_2_O_2_ level was measured. Values are means ± SD for at least three independent experiments performed in triplicate (*P < 0.05 and **P < 0.01 compared with vehicle control).

To further study the effect of the measured complexes on cell cycle arrest, PI staining assays were performed to analyze the cell cycle distribution of NSCLC cells treated with oxaliplatin, complex 3 and complex 3a at 10 μM. The results in Figure 3A shown that the G2/M phases of A549 and NCI-H1299 were blocked strikingly as exposed to complex 3 compared to complex 3a, while S phases were blocked as treated by oxaliplatin. With the untreated A549 and NCI-H1299 cells, the percentage of cells in the G1 phase was at 21.29%, 18.96% with 15.28%, 22.94% in the G2/M phase, respectively. After treatment with complex 3, the percentage of cancer cells in the G1 phase increased to 57.85% and 60.29%. The cell cycle arrest induced by complex 3 compared favorably to the cells treated with oxaliplatin. The results also indicated that the large steric hindrance of complex 3 could increase cell cycle arrest as compared to its mono-substituted complex 3a.

To further study the detailed process of apoptosis induced by complex 3 in NSCLC cells, we examined the mitochondrial membrane potential (ΔΨm), which is an important proapoptotic index for early apoptosis. We chose a mitochondria-specific and voltage-dependent fluorescent probe, JC-1, to observe whether there was loss in ΔΨm. The results in Figure 3B, exposure to complex 3 of 10 μM for 24 h resulted in elevation in the percentage of cells with green fluorescence in A549 and NCI-H1299 cancer cells, which indicated a remarkable decrement of ΔΨm in complex 3-treated cells. The results from flow cytometry shown that complex 3 could make ΔΨm decrease mostly compared with oxaliplatin (Figure 3C).

Since ATP production is a significant consequence attributed to the dysfunction of mitochondrial energy production, we measured intracellular ATP levels after complex 3 treatment. As shown in Figure 3D, the ATP level in cells treated with complex 3 at 10 μM, decreased to 19.95%, 31.89% of untreated A549 and NCI-H1299 cancer cells, respectively, which is 28.62% and 22.71% lower than that of oxaliplatin-treated cancer cells.

### Complex 3 increased ROS and H_2_O_2_ generation of NSCLC cells

As excessive ROS generation is associated with cell-killing activity [31], we further examined whether complex 3 could also induce the formation of excessive ROS in A549 and NCI-H1299 cancer cells. Cancer cells were exposed to 10 μM oxaliplatin, complex 3 and complex 3a and then fluorescence intensity was detected in response to the levels of ROS using DCF-DA by flow cytometry. As shown in Figure 3E, fluorescence intensity was increased rapidly in complex 3-treated groups, indicating that complex 3 induced the production of significant amounts of ROS in A549 and NCI-H1299 cancer cells. Taking into account that among all ROS and other oxygen-derived free radicals, H_2_O_2_ has been recently suggested to act as a central player in signal transduction pathways [32], we evaluated H_2_O_2_ generation by a fluorometric assay in cancer cells incubated in the presence of 10 μM oxaliplatin, complex 3 and complex 3a. H_2_O_2_ measurement was carried out after 2 h incubations with oxaliplatin, complex 3 and complex 3a, respectively. The production of H_2_O_2_ was significantly higher in cancer cells incubated with complex 3 than that in cells treated with complex 3a and oxaliplatin in A549 and NCI-H1299 cancer cells (Figure 3F). The results strongly suggested that complex 3 could induce increased intracellular oxidative stress compared with complex 3a and oxaliplatin, thus triggering NSCLC cancer cell apoptotic pathways.

### Complex 3 induced ROS-mediated DNA damage in A549 cells

**Figure 4:**
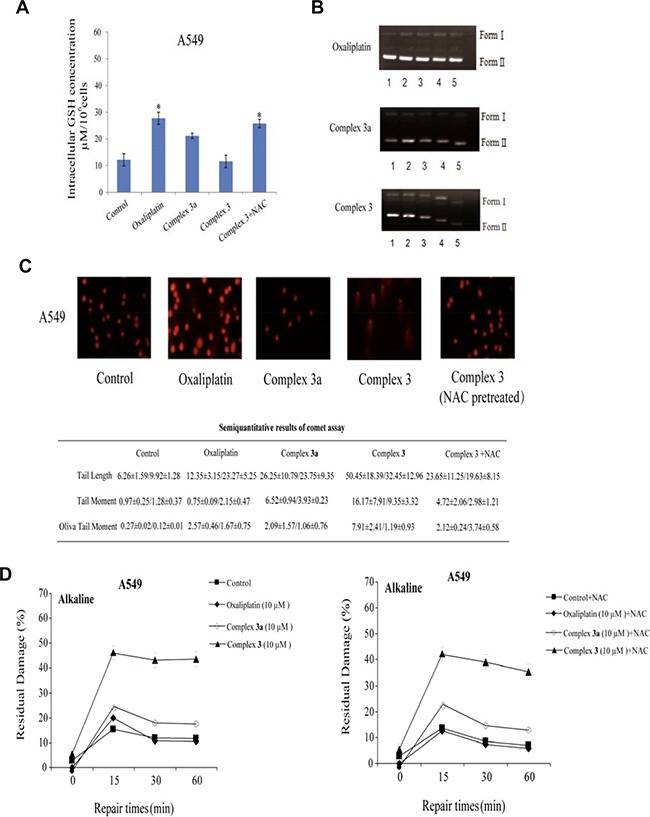
Intracellular GSH concentrations and DNA damage of the measured complexes induced SSBs and DSBs (**A**) A549 cells were pretreated with NAC for 30 min and then incubated with oxaliplatin, complex 3 and complex 3a (10 μM) for 4 h. (**B**) Gel electrophoretic mobility pattern of pET28a plasmid DNA incubated with various concentrations of platinum(II) complexes. Lanes 1–5 (0, 20, 80, 320, 640 μM) + DNA. A) oxaliplatin; B) complex 3a; C) complex 3. (**C**) Comet assay revealing increased chromosomal DNA strand breaks including SBBs, DSBs and active excision repair of DNA cross-links triggered by the measured complexes in A549 cancer cells with or without NAC pretreatment. (**D**) The number of SBBs was determined by a neutral comet assay with or without NAC pretreatment. Graph represents average number of foci per cells ± SD. Results are representative of at least three independent experiments and shown as the mean ± S.D. *P < 0.05, **P < 0.01 compared with control group.

Following the complex 3 treatment, we measured intracellular GSH levels in A549 cancer cells. As shown in Figure 4A, while treating with complex 3, however, intracellular GSH levels did significantly reduce, which could be ceased by NAC treatment. These results proved that complex 3 reduces intracellular GSH levels to improve active platinum delivery to the DNA to interact with cellular components and causes severe DNA damage [33, 34]. Agarose gel electrophoresis was applied to study the interaction of pET28a plasmid DNA with oxaliplatin, complex 3a and complex 3. The tested complexes were incubated with DNA at different concentrations. In the electrophoretogram (Figure 4B), untreated pET28a plasmid DNA mainly consisted of covalently closed circular (Form I) and a small amount of nicked (Form II) bands. Owing to the unwinding of pET28a plasmid DNA, a decrease in the rate of migration for closed circular DNA (Form I) was observed for oxaliplatin. Moreover, a coalescence of the closed circular DNA (Form I) and open circular DNA (Form II) was observed for complex 3, indicating a strong unwinding of the supercoiled DNA. However, no migration was observed for complex 3a, which is probably due to the fact that the large steric hindrance of complex 3 greatly increases the interaction between the complex and DNA.

### Complex 3 suppressed the activation of PI3K/Akt/mTOR signaling pathways in autophagic cells death

**Figure 7:**
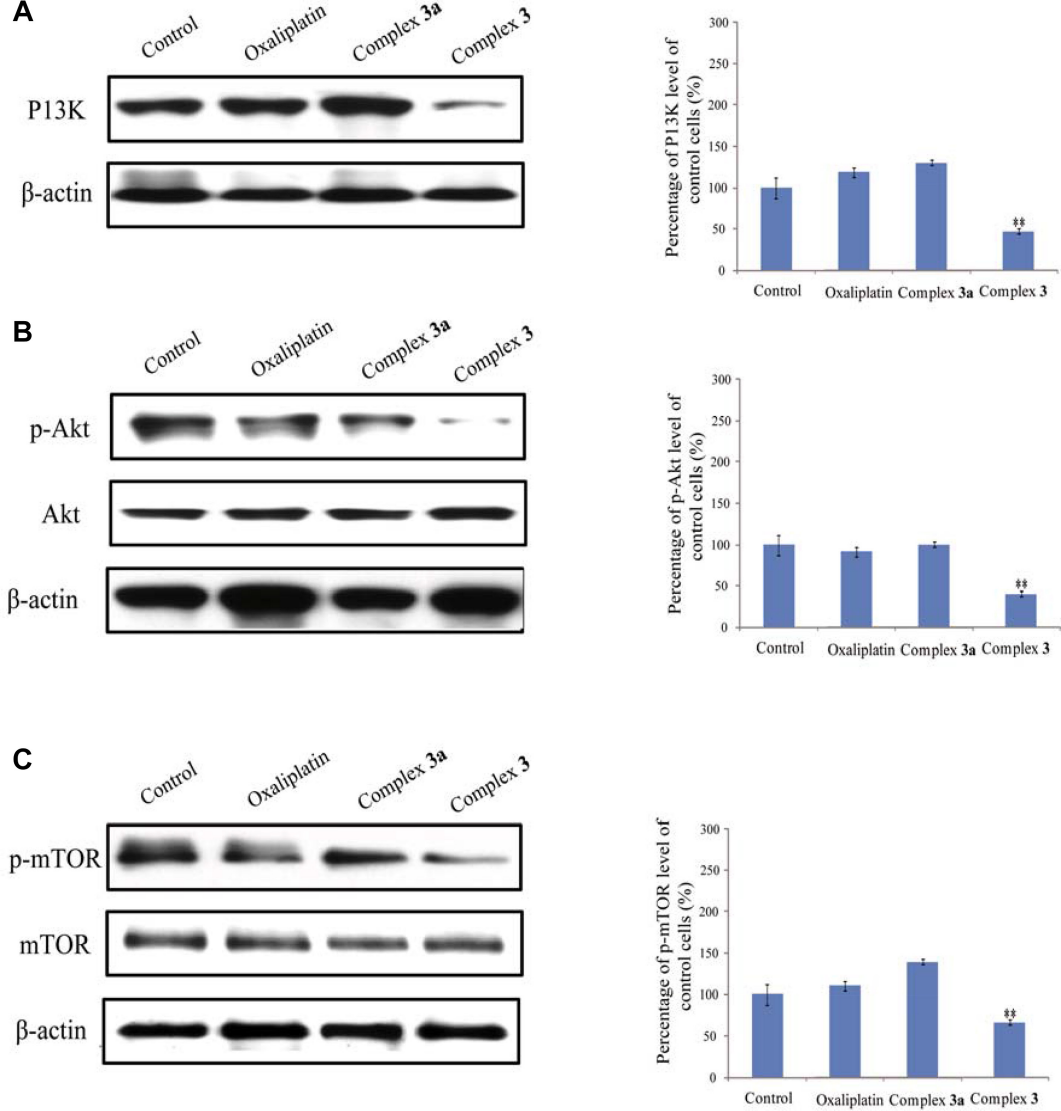
Complex 3 inhibits the PI3K/Akt/mTOR signaling pathway Human A549 cells were exposed to 10 μM complex 3 for 24 h. (**A**) Levels of PI3K was immunodetected. β-Actin was detected as an internal control. These protein bands were quantified and statistically analyzed. (**B** and **C)**, Levels of p-Akt and p-mTOR were immunodetected. Akt, mTOR, and β-actin were detected as the internal controls. These immunorelated protein bands were quantified and statistically analyzed. Each value represents the mean ± SEM from three independent experiments. The symbols * indicate that values significantly (*P < 0.05, **P < 0.01) differed from the respective control group.

The mammalian target of rapamycin (mTOR) kinase is used to regulate autophagy as controlled though oxidative stress, hypoxia and growth receptors [36]. The phosphatidylinosital-3 kinase (PI3K)/Akt signaling pathway activates mTOR through phosphorylating and inactivating the tuberous sclerosis complex (TSC)1–TSC2 complex [37]. Inhibition of mTOR results in activation of autophagy [38]. Thus, treatment of A549 cells with 10 μM oxaliplatin, complex 3 and complex 3a for 24 h inhibited the protein levels of PI3K to 1.01-, 1.25- and 0.42-folds, respectively (Figure 7A). At the same time, levels of the p-Akt protein decreased to 0.95-, 1.02-, and 0.39-folds, respectively (Figure 7B). Moreover, amounts of p-mTOR were also decreased to 1.15-, 1.41-, and 0.69-folds (Figure 7C). As a results, the administration of complex 3 decreased levels of proteins in PI3K/Akt/mTOR signaling pathways.

As a key role in oxidative stress, ROS can directly interact with cellular components and causes severe DNA damage [34, 35]. Thus, we further identified whether the increased ROS level serves as a cause of DDR. Based on comet assay, our data showed a proportionate increase in three major parameters of comet assay (tail moment, olive tail moment and tail length) as time passed in complex 3-treated cells, whereas these indicators showed minor changes when cells were pre-treated with NAC (Figure 4C). To investigate the extent of DNA damage induced by complex 3, we detected the numbers of normal SSB using an alkaline elution assay. The results observed in cancer cells treated with the tested complexes at 10 μM in 15, 30 and 60 min indicated that complex 3 could effectively increase the numbers of SSBs, which could not be inhibited by NAC treatment (Figure 4D). However, complex 3 was found to induce higher DNA damage levels of SSBs than oxaliplatin and complex 3a, implying that with the time-dependent DNA repair response of SSBs, the increased DNA damage level happened in complex 3-treated cancer cells.

### Complex 3 induced ROS-mediated DNA double strand breaks and blocked DNA repair response in A549 cells

**Figure 5:**
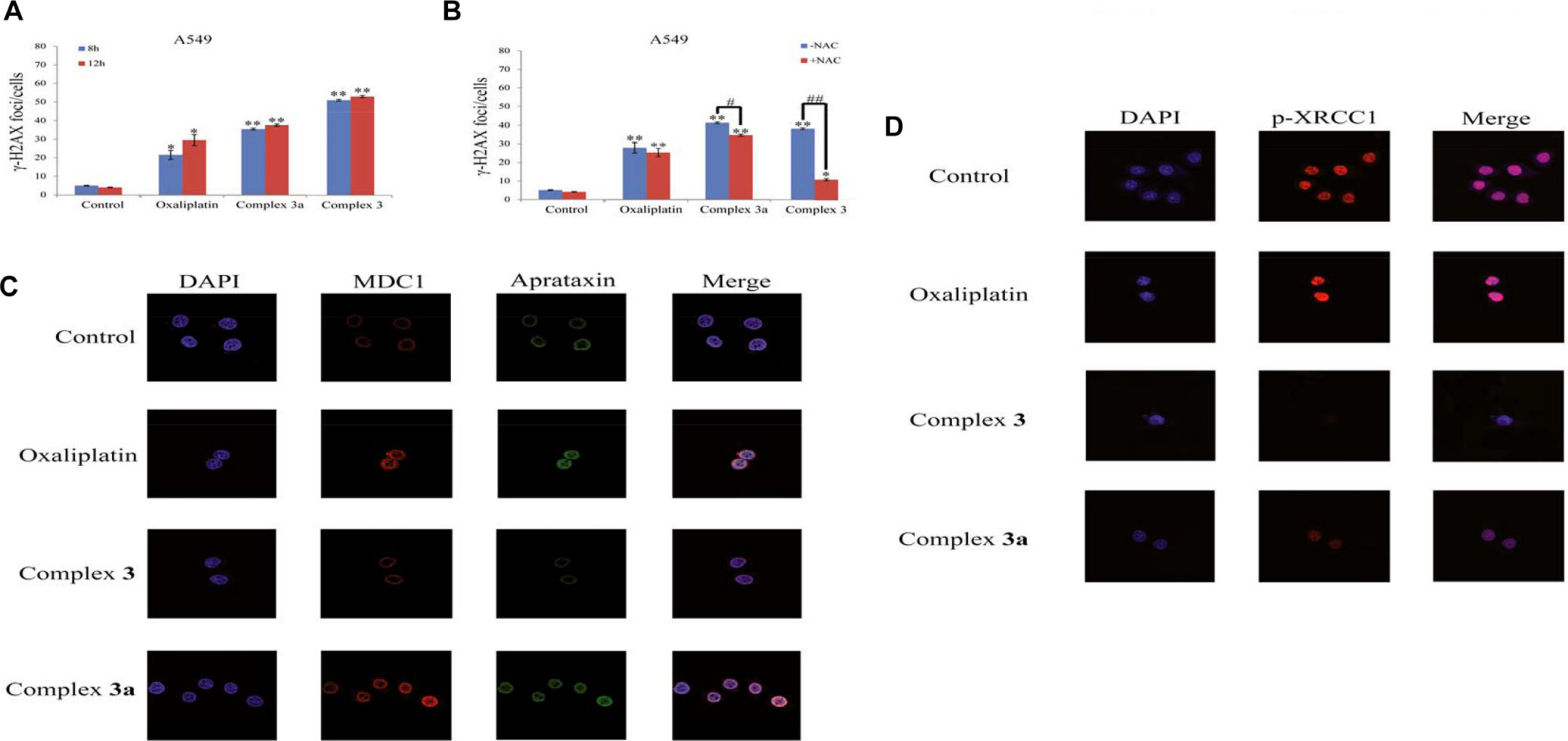
The inhibitory effect of the measured samples on DNA repair response (**A**) γH2AX foci after treatments were counted in 50–60 individual cells of 8 h and 12 h in A549 cells. (**B**) γH2AX foci after pretreatment with NAC were counted in 50–60 individual cells of 12 h in A549 cells. (**C**) Complex 3 could inhibit the recruitment of DSB repair proteins MDC1 and Aprataxin to damaged chromatin. (**D**) The inhibitory effect of the measured samples on DNA single strands repair response. A549 cancer cells were treated with the measured complexes at 10 μM for 24 h. After that, cells were pre-extracted with detergent, fixed and immunostained with antibodies against MDC1/Aprataxin and p-XRCC1. Results are representative of at least three independent experiments and shown as the mean ± S.D. *P < 0.05, **P < 0.01 compared with control group.

The serine 139-phosphorylated form of chromatin-associated histone H2AX, namely γ-H2AX, is a key marker of cellular response to DDR, particularly DSBs. We detected obvious increases in γ-H2AX when cells were exposed to complex 3 (Figure 5A). Obviously, pre-treatment of NAC could considerably alleviate the DDR triggered by complex 3 (Figure 5B). Therefore, we came to a conclusion that complex 3 caused DDR especially DSBs in A549 cells, which might happen after the increased level of intracellular ROS. In order to reveal the mechanisms underlying the enhanced DNA damage, complex 3 was studied to learn whether it could reduce the activation of DNA repair response including the co-localization of DSB repair protein MDC1/Aprataxin within nucleus. Treatment of A549 cells with 10 μM complex 3 for 24 h led to a dramatically decrease in the co-localization of MDC1/Aprataxin to the damaged DNA (Figure 5C). The different fluorescence intensities of MDC1/Aprataxin induced by oxaliplatin, complex 3 and complex 3a, respectively, indicated that DSBs happened mostly in the treatment with complex 3. In order to reveal the mechanisms underlying DNA damage, complex 3 was studied to detect whether it could suppress the activation of DNA single strand break repair via the phosphorylation of SSB repair mediator/adaptor proteins XRCC1 within nucleus. Treatment of A549 cells with 10 μM complex 3 for 24 h resulted in the suppression of phosphorylation of XRCC1 compared with oxaliplatin (Figure 5D). The results revealed that complex 3 could inhibit extensive DNA damage repair in addition to its ability of increasing cellular Pt uptake.

### Complex 3 induced autophagy in cells death

**Figure 6:**
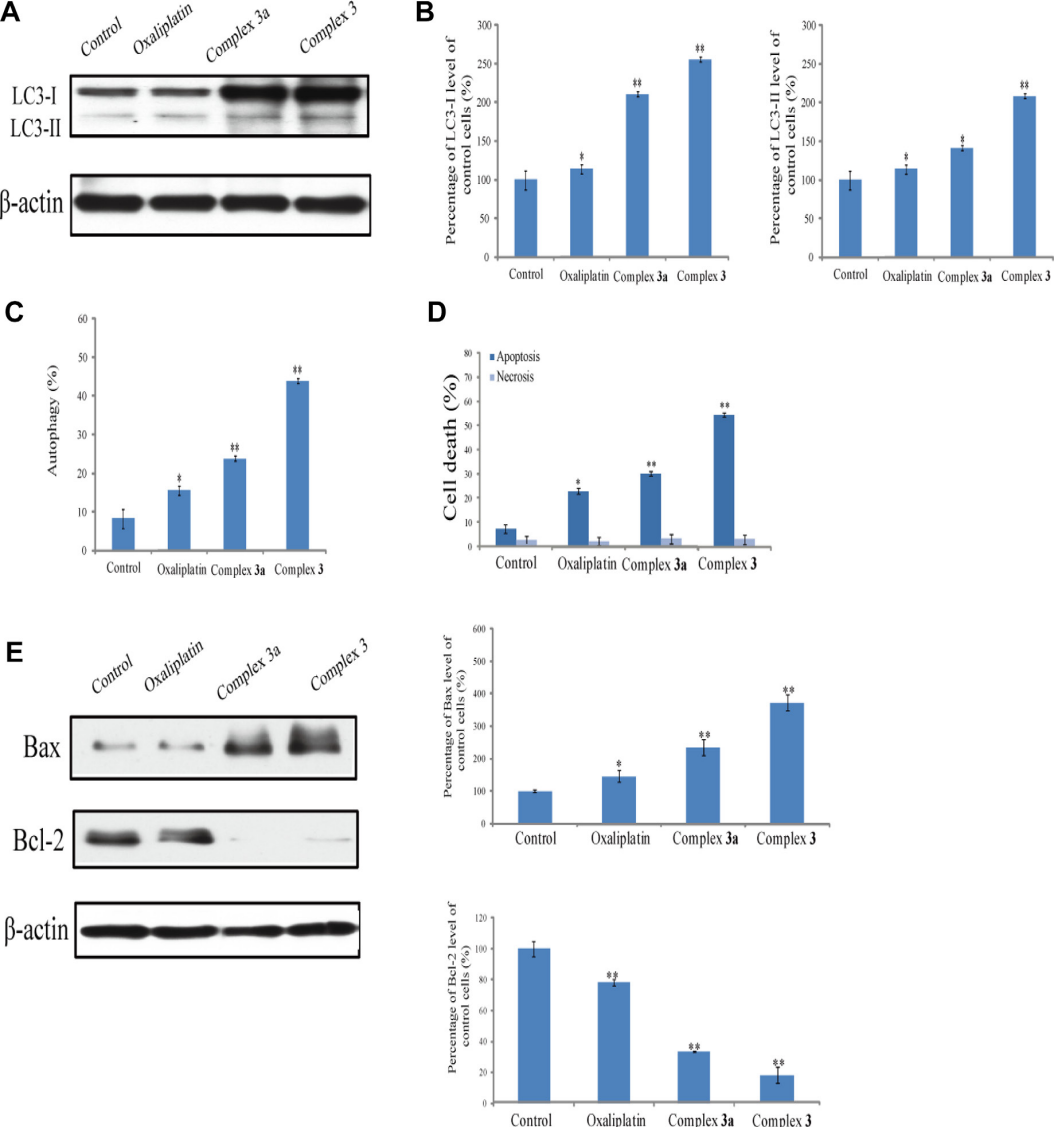
Complex 3 induces autophagic cell death Human A549 cells were treated with measured complexes of 10 μM for 24 h. (**A**) Levels of LC3 were immunodetected. β-actin was detected as the internal control. (**B**) These protein bands were quantified and statistically analyzed. (**C**) The percentage of autophagy was quantified using flowcytometry. (**D**) Cell apoptosis was quantified using flow cytometry. (**E**) Bax and Bcl-2 levels in the whole-cell lysates. Each value represents the mean ± SEM from three independent experiments. The symbols * indicate that values significantly (*P < 0.05, **P < 0.01) differed from the respective control group.

The role of complex 3-induced autophagy in cell death was further analyzed (Figure 6). When A549 cancer cells were treated with complex 3 of 10 μM, the protein expression of LC3-II was increased (Figure 6A). Compared to the complex 3-treated group, exposure of A549 cells to oxaliplatin and complex 3a of 10 μM decreased the level of LC3-II from 2.5- to 1.3-folds (Figure 6B). Treatment with complex 3 for 24 h caused a 4.5-folds increase in cells undergoing autophagy (Figure 6C). In contrast, when A549 cells were treated with 10 μM oxaliplatin and complex 3a, the number of autophagic cells was decreased. After A549 cells treated with oxaliplatin, complex 3a and complex 3 for 24 h, the percentage of autophagic cell apoptosis decreased from 21.9% to 41.5% (Figure 6D). Subsequently, complex 3 resulted in a decrease in cell viability. Moreover, Bax and Bcl-2 expressions were measured by western blot analysis of whole cell lysates. Incremental changes in pro-apoptotic Bax and decreased changes anti-apoptotic Bcl-2 protein levels were also observed (Figure 6E), suggesting that complex 3 may modulate apoptosis in A549 lung cancer cells.

### Complex 3-induced autophagy were mediated by ROS in A549 cells

**Figure 8:**
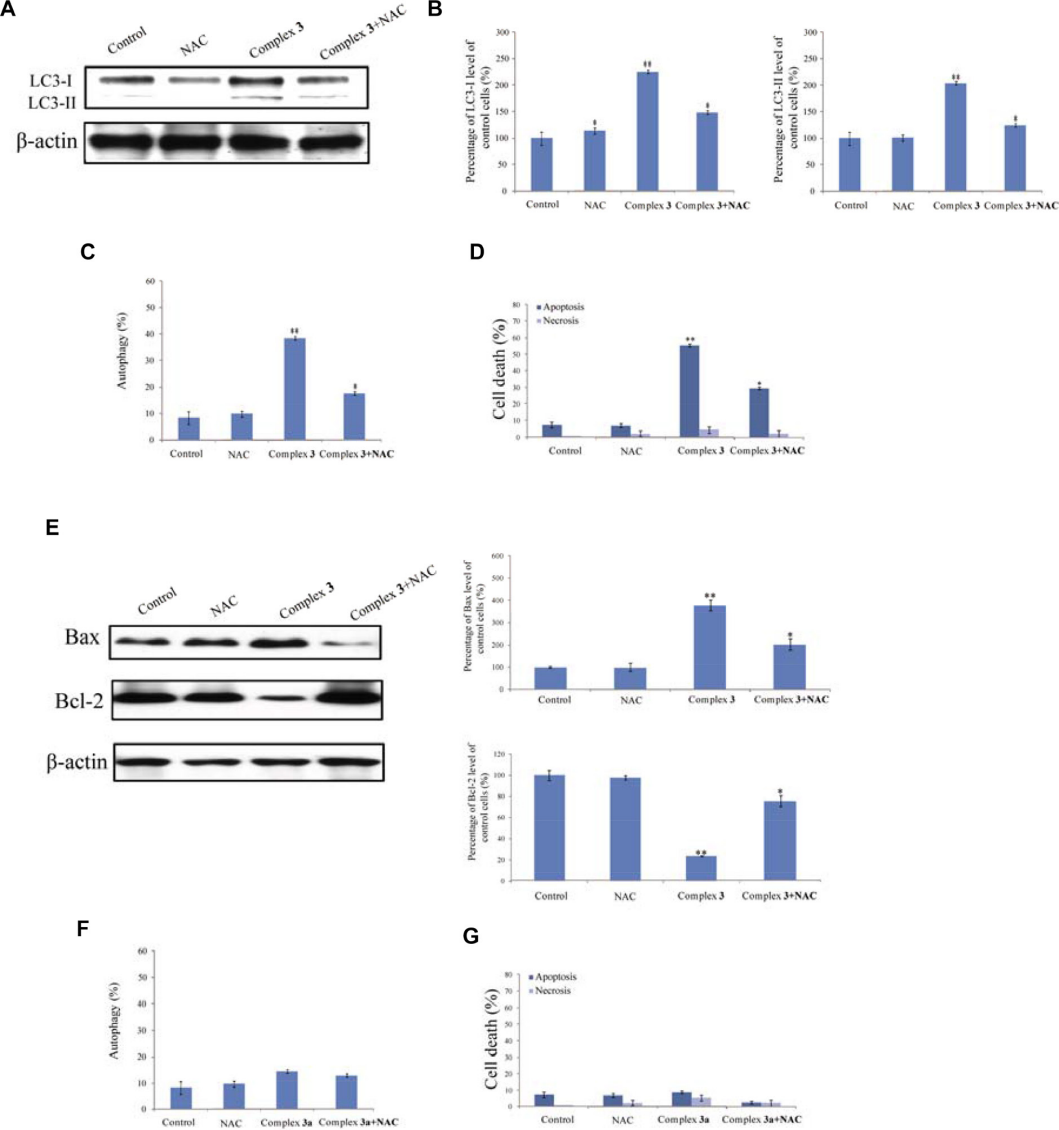
Reactive oxygen species (ROS) are involved in complex 3-induced autophagic death (**A**) A549 cells were pretreated with 5 mM NAC for 2 h and then with 10 μM complex for another 18 h. Levels of LC1 and LC3 were immunodetected. β-Actin was immunodetected as the internal control. (**B**) These protein bands were quantified and statistically analyzed. The percentage of autophagy (**C**) and cell viability (**D**) were analyzed using flow cytometry. (**E**) Levels of Bax and Bcl-2 were immunodetected. β-Actin was used as the internal control. The protein bands were quantified and analyzed. (**F**) The percentage of autophagy was quantified using flow cytometry following the pretreatment with NAC in complex 3a-treated A549 cells. (**G**) Cell apoptosis was quantified using flow cytometry with NAC pretreatment in complex 3a-treated A549 cells. Each value represents the mean ± SEM from three independent experiments. The symbols * indicate that values significantly (*P < 0.05, **P < 0.01) differed from the respective control group.

To investigate the role of ROS in complex 3-induced cell death, the level of intracellular H_2_O_2_ was measured. After exposure of A549 cancer cells to 10 μM complex 3, the increased level of the LC3-II protein was also reduced by NAC (Figure 8A). Exposure of A549 cells to NAC reduced levels of LC3-II from 2.3- to 1.5-folds (Figure 8B). Consequently, pretreatment with NAC decreased the percentage of autophagy from 42.5% to 11.8% (Figure 8C), and the percentage of apoptosis from 52.5% to 34.2% (Figure 8D). In parallel, the complex 3-induced increase ratio of Bax/Bcl-2 was suppressed by NAC (Figure 8E). Moreover, pretreatment with NAC in complex 3a-treated A549 cancer cells showed no decreased percentage of autophagy (Figure 8F) and apoptosis (Figure 8G).

### Complex 3 inhibited tumor growth and autophagy in the mice xenograft model

**Figure 9:**
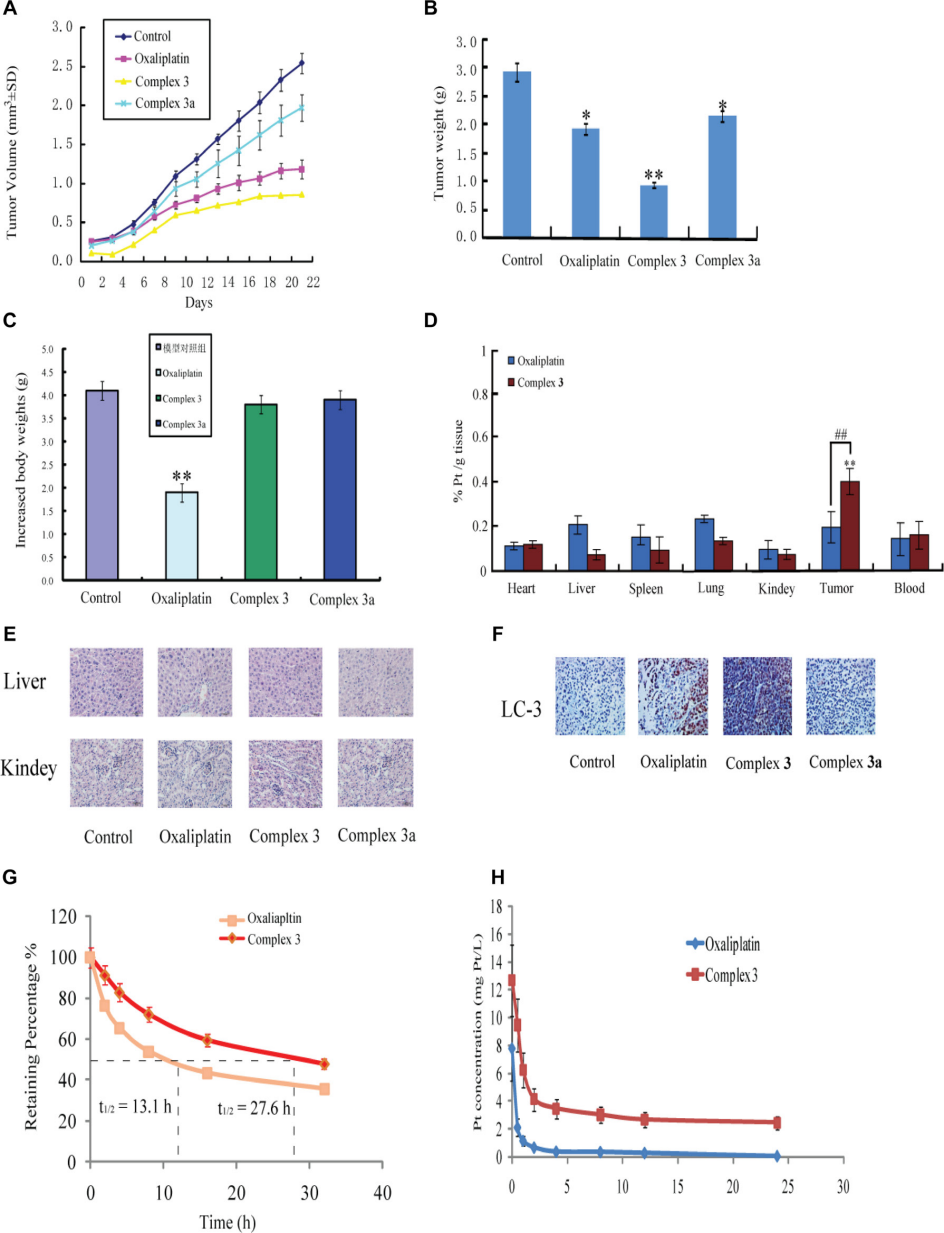
*In vivo* antitumor activity of oxaliplatin, complex 3 and complex 3a in A549 xenograft tumors Mean tumor volumes, changes in tumor and body weights are presented. Oxaliplatin (dosed intravenously at 5 mg/kg twice a week), complex 3 (dosed intravenously at 5 mg/kg once every three days), complex 3a (dosed intravenously at 5 mg/kg once every three days). (**A**) The tumor growth in xenograft mouse models at the administration of the corresponding groups. (**B**) The tumor weight in each group at the end of the experiment. (**C**) Measured weight increase of mice during the treatments. (**D**) Tissues and tumor distribution of cisplatin and complex 3 in mice bearing A549 tumors after i.v. administration of the corresponding groups. Major organs were collected at 1 h after injection. (**E**). The HE staining of normal tissues of Liver and Kindey. (**F**) immunoblotting analysis of LC3. (**G**) Stability of oxaliplatin and complex 3 in rat plasma. (**H**) *In vivo* pharmacokinetic curves of Pt concentration in the rat plasma versus time after intracenous injection of cisplatin and complex 3 in rats. Results are representative of at least three independent experiments and shown as the mean ± S.D. *P < 0.05, **P < 0.01 compared with control group.

To evaluate the anti-tumor effect of complex 3 *in vivo*, an xenografts nude mice model of A549 cancer cells was performed. Oxaliplatin (dosed intravenously at 5 mg/kg twice a week), complex 3 (dosed intravenously at 5 mg/kg once every three days), complex 3a (dosed intravenously at 5 mg/kg once every three days) were conducted into seven nude mice groups. There was no any death during the tumor formation in nude mice. The statistical results indicated that the tumor volumes (Figure 9A) in complex 3-treated group were significantly decreased compared to the same dosage of cisplatin-treated group. At the end of treatments, the tumor weight was measured to detect the antitumor activity of these measured groups. The tumor growth inhibition rate of complex 3 reached 67.96%, while that of oxaliplatin at the same dose as complex 3 was only 34.48% (Figure 9B). Because platinum-based drugs are well known for their toxic effects on the mean bodyweights of treated animals, the toxicity of complex 3 in the body weights of mice was assayed. The results in Figure 9C illustrated that the increase of bodyweights was observed in the treatment of complex 3, however, oxaliplatin exerted a continuous decrease of bodyweights. The mice treated with the tested complex 3 were selected for histological analyses. The side effects of complex 3 decreased significantly can be explained by the *in vivo* distribution behaviors. The level of Pt in liver, spleen, lung and kidney were significantly reduced as the drug tumor distribution was significantly increased when mice were injected with complex 3 (Figure 9D). The HE staining of slices from liver and kidney demonstrated that complex 3 had hardly toxic effect on normal tissues in contrast to cisplatin (Figure 9E). After administration of measured complexes, complex 3 increased expressions of LC3 in tumor tissues (Figure 9F) which the *in vivo* effect of complex 3 on inducting autophagy was also evaluated. These data proved that complex 3 exhibits pronounced antitumor activity with nearly no toxic effect on treated animals in contrast to oxaliplatin and complex 3a.

### Pharmacokinetics

The stability of complex 3 in blood is significantly important for its clinical application. Thus, we investigated the stability of complex 3 in rat plasma via ICP-MS measurement. Fresh rat plasma was added with a DMSO solution of complex 3 (10 μM). After incubation for 0, 2, 4 and 8 h at 37°C, the Pt content in rat plasma was measured by ICP-MS. The results in Figure 9G reveals the half-life of complex 3 in rat plasma to be 27.6 h, which is significantly longer than that of oxaliplatin (t_1/2_ ~ 13.1 h). This interaction contributes to the promising stability of complex 3 in rat plasma.

The pharmacokinetic profile of complex 3 was performed as compared with cisplatin. The results showed that cisplatin and complex 3 followed a two-compartment pharmacokinetic model, which was based on the residual sum of squares and minimum Akaike's information criterion (AIC) value. A non-compartment model was applied to analyze changes of pharmacokinetic behaviors. The results in Figure 9H showed the Pt concentration-time profiles in rat plasma, revealing the half-life of complex 3 (t_1/2_~ 24.5 h) to be 3.61-folds increased compared to oxaliplatin (t_1/2_~ 13.1 h). Although rapid elimination of Pt via blood circulation occurred in both cisplatin and complex 3 groups, changes of these preparations were significantly different and significant increased release time happened in the complex 3-treated group. Complex 3 had a longer blood retention as compared to cisplatin, which could dramatically promote Pt accumulation in tumor tissues in complex 3-treated group. The results could be attributed to the biodistribution of the complex 3 in normal tissues as well as retention in blood circulation. Thus, complex 3 with longer circulation time could significantly alleviate *in vivo* pharmacokinetics of cisplatin and increase its bioavailability.

Upon above all the study, complex 3, possessing significant antitumor activity via inhibiting oxidative DNA damage and autophagic responses and exhibiting nearly no toxic effect, has a potential promise to be a platinum-based anticancer drug candidate.

## DISCUSSION

Platinum-based chemotherapeutics are widely used clinically via causing most human tumor regression. However, the DNA-damaged platinum-based drugs have been intensively studied for their antitumor efficacy with high levels of DNA adducts lesions, finally causing cell cycle arrest and cell death, and thus have severe toxicity and acquired resistance. Our group has previously designed a series of platinum-based complexes bearing N-mono-substituted 1*R*,2*R*-diaminocyclohexane derivatives as carrier ligands which showed optimistic antitumor activity compared with oxaliplatin. More important, we synthesized several Pt(II) complexes of (1*R*,2*R*)-N^1^,N^2^-dibutyl-1,2-diaminocyclohexane with two n-butyl branches as steric hindrance. The data showed that the increase of sterically hindered effect of the platinum complexes could improve the cytotoxic activity and decrease the side effects, thus we intend to introduce two alkyl moieties to the 1*R*,2*R*-DACH skeleton to further study the sterically hindered effect of the platinum complexes. The study proved that complex 3 could induce oxidative DNA damage in NSCLC cells. In a continuation of those results, herein we revealed that complex 3 can induce autophagy of A549 cells *in vitro and in vivo*. Moreover, this study showed that suppression of ROS production could inhibit DNA damage and autophagic responses, which could consequently lower apoptosis of human lung cancer cells. Therefore, complex 3 can induce apoptosis and autophagic death of cancer cells. Apoptosis and autophagy are classified as programmed cell death and there is an interconnection between autophagy and apoptosis [39], which could result in cell death and cooperate during this process [40]. For instant, autophagy was reported to result in traumatic brain injury-induced apoptosis [41]. In this study, inhibition of autophagy by NAC decreased apoptosis and reversed cell viability. Our results suggest that complex 3-induced autophagy contributes to apoptotic cell death. On the contrary, pro-survival autophagy also occurred in some models to reduce apoptosis, such as imatinib-treated gastrointestinal stromal tumor cells and cisplatin-treated gastric cancer cells [42, 43]. Thus, the role of complex 3-induced autophagy in different cancer cells is still controversial.

Previous studies proved that the PI3K/Akt/mTOR signaling pathway is involved in autophagy induction [43, 44]. Herein, after treating with complex 3, whether autophagy was induced and was accompanied by reduced protein levels of PI3K, p-Akt and p-mTOR should be further studied. Additionally, hypoxia has been shown to regulate autophagy of cancer cells [45]. Previous stuides reported that a combined treatment with celecoxib and γ-irradiation could synergistically induce autophagy of hypoxic cancer cells [46]. Thus, hypoxia may be another critical factor that can synergistically induce autophagy of cancer cells with complex 3. In addition to cell cycle arrest, senescence, and apoptosis, p53 can stimulate autophagy [47]. Activation of p53 results in upregulation of unc-51-like kinase 1/2, which is necessary for sustained autophagy in response to camptothecin-induced DNA damage [48]. However, determining how p53 regulates autophagy after complex 3 treatment requires further investigation.

Numerous studies have demonstrated that increased ROS stress in cancer cells leads to apoptosis [49]. However, other studies indicated that ROS participate in autophagy to regulate cell survival. A novel celecoxib derivative, OSU-03012, induced ROS-related autophagic cell death in hepatocellular carcinoma [50]. Silibinin triggered ROS generation, including H_2_O_2_ and O^2·−^, to induce autophagic and apoptotic cell death of HT1080 human fibroblast cells [51]. In this study, ROS and H_2_O_2_ were generated after treatment with complex 3 and were suppressed by NAC, resulting in a decrease in autophagic and apoptotic cell death of A549 cells. Our results showed that complex 3-induced autophagy contributes to A549 cancer cell death through ROS generation. This study suggests that ROS may cause cell death via apoptosis and autophagy. However, the role of ROS-induced autophagy in cell death is still controversial. DNA damage induced by ROS and chemotherapeutic agents promotes p53 activation, which causes caspase-dependent apoptosis and poly (ADP-ribose) polymerase-1-mediated necrosis [52]. Those studies showed that interlinks between ROS and p53 are complex, and outcomes may be determined by various stimuli or cell types.

In our present study, we designed, synthesized and characterized a series of platinum(II) complexes with (1*R*,2*R*)-N^1^,N^2^-diisobutyl-1,2-diaminocyclohexane as a carrier ligand and chloride and dicarboxylates as leaving groups. The *in vitro* cytotoxicity study revealed that complex 3 had significant cytotoxicity effect against all the tested cell lines compared to its mono-substituted complex 3a, indicating that the function of alkyl moieties, as steric hindrance, has a significant influence on the antitumor property of the resulting platinum complexes. The flow cytometric assay indicated that complex 3 could induce increased apoptotic rate and ROS generations of cancer cells compared to oxaliplatin. The comet assay and γH2AX foci assay proved its activity to induced DNA damage including double strand breaks obviously, which could be ceased by ROS scavenger NAC in A549 cells. Meanwhile, our results proved for the first time that treating A549 cells with complex 3 induced ROS-mediated autophagic cell death. Additionally, NAC, an antioxidant, improved cell survival by inhibiting autophagy and apoptosis, which indicates the important role of ROS in regulating complex 3-induced cell death. Therefore, this study showed the unique structure of large steric hindrance could enhance its antitumor activity higher compared to oxaliplatin via ROS-mediated DNA damage and autophagic cell death. Consequently, the introduction of appropriate alkyl moieties to the 1*R*,2*R*-DACH skeleton as steric hindrance can be a candidate to design platinum complexes in future study.

## MATERIALS AND METHODS

### Materials and instruments

All chemicals and solvents were of analytical reagent grade and were used without further purification. Potassium tetrachloroplatinate(II) was obtained from a local chemical company. The ligand was prepared by the method as described previously in our laboratory. Silver dicarboxylates were prepared by the reaction of the corresponding sodium dicarboxylate with silver nitrate in water. Infrared spectra were measured on KBr pellets on a Nicolet IR200 FT-IR spectrometer in the range of 4000–400 cm^−1^. ^1^H NMR spectra were recorded in *d*_6_-DMSO with a Bruker 300MHz NMR spectrometer. Mass spectra were measured on an Agilent 6224 TOF LC/MS instrument.

### Synthesis and xharacterization

### Synthesis of complex 1

A solution (20 mL) of K_2_PtCl_4_ (2.1 g, 5.0 mmoL) was added to the ligand (1.1 g, 5.0 mmoL) in water (5 mL). The reaction mixture was heated to 60°C and stirred for 72 h in the dark. Plenty of yellow precipitate was filtered off, washed with water repeatedly, and then dried in vacuum.

### General synthesis of complexes 2–4

To a suspending aqueous solution (150 mL) containing 1 mmoL of complex 1, silver dicarboxylate (1 mmoL) was added. The reaction mixture was heated to 40°C and stirred for 24 h under the lighting shielding condition. After the mixture was cooled to the room temperature, AgCl deposits were filtered off and washed with water. The filtrate was concentrated to 5–10 mL by a rotatory evaporator and then kept cool at 4 °C for several hours. The resulting off-white solids were filtered off, washed with a small quantity of iced water, and then dried in vacuum.

### Complex 1

(C_14_H_30_Cl_2_N_2_Pt): Yield:95%, yellow powder. Elem anal. Calcd for C_14_H_30_Cl_2_N_2_Pt: C, 34.15; H, 6.11; N, 5.70. Found: C, 34.33; H, 6.36; N, 5.48. IR (KBr, cm^−1^): 3444(br), 3121,2950,2863,1459, 1235,1159,505;^1^H NMR (*d_6_-*DMSO/TMS, ppm): δ1.07–1.18 (m, 12H, CH*CH_3_*), 1.21–1.90 (m, 8H, *CH_2_*of DACH), 2.21–2.30 (m, 2H, *CH*CH_3_), 2.63–2.69 (m, 2H, *NH*CH),2.85–2.90 (m, 4H, NH*CH_2_*), 4.58 (m, 2H, *NH*); ESI-MS: m/z[M+Cl]^−^= 526(100%).

### Complex 2

(C_16_H_30_N_2_O_4_Pt): Yield: 51%, white powder. Elem anal. Calcd for C_16_H_30_N_2_O_4_Pt: C, 37.72; H, 5.93; N, 5.50. Found: C, 37.53; H, 5.68; N, 5.25. IR (KBr, cm^−1^): 3860(br), 3147, 2955, 1675, 1462, 1396, 805; ^1^H NMR (*d_6_-*DMSO/TMS, ppm): 0.90–1.06 (m, 12H, *CH_3_*), 1.33–1.97 (m, 10H, *CH_2_*of DACH and CH_3_*CH*), 2.11–2.21 (m, 4H, NH*CH_2_*), 2.57 (m, 2H, NH*CH*), 6.50 (s, 2H, CH*NH*); ESI-MS: m/z [M+Na]^+^= 532(100%).

### Complex 3

(C_17_H_32_N_2_O_4_Pt): Yield: 21%, white powder. Elem anal. Calcd for C_17_H_32_N_2_O_4_Pt: C, 39.00; H, 6.12; N, 5.35. Found: C, 38.81; H, 6.01; N, 5.15. IR (KBr, cm^−1^): 3399(br), 1579, 1355, 696; ^1^H NMR (*d_6_-*DMSO/TMS, ppm): 0.99–1.02 (m, 12H, *CH_3_*), 1.12–1.63 (m, 10H, *CH_2_*of DACH and CH_3_*CH*), 2.06–2.12 (m, 4H, NH*CH_2_*), 2.24–2.33 (m, 2H, NH*CH*), 3.19 (s, 2H, (CO)_2_*CH_2_*), 6.12 (m, 2H, CH*NH*); ESI-MS: m/z [M+Na]^+^= 546(100%).

### Complex 4

(C_20_H_36_N_2_O_4_Pt): Yield: 33%, white powder. Elem anal. Calcd for C_20_H_36_N_2_O_4_Pt: C, 42.63; H, 6.39; N, 4.97. Found: C, 42.75; H, 6.47; N, 4.72. IR (KBr, cm^−1^): 3122(br), 2951, 2868, 1646, 1463, 1350,1114, 515; ^1^H NMR (*d6-*DMSO/TMS, ppm): 0.89–1.03 (m, 12H, *CH3*), 1.05–1.70 (m, 10H, *CH2*of DACH and CH_3_*CH*), 2.07–2.11 (m, 2H,*CH*_2_ of cyclobutyl), 2.23–2.36 (m, 4H, NH*CH2*), 2.61–2.67 (m, 2H, NH*CH*), 2.70–2.76 (m, 4H,*CH*_2_ of cyclobutyl), 5.85 (m, 2H, *NH*); ESI-MS: m/z [M+Na]^+^ = 586(100%).

### Cell culture

Human cancer cell lines HepG2, SGC-7901, HCT-116, A549, NCI-H1299 and MCF-7 as well as normal liver cell line L02 were purchased from the Cell Bank of Shanghai Institute of Cell Biology. Among them, MCF-7, HepG2 cells were cultured at 37 °C in 5% CO_2_ with DMEM supplemented with 10% fetal bovine serum (FBS) (Hyclone, Lifescience, USA) and 1% penicillin/streptomycin (Beyotime, Nantong, China), while HCT-116, SGC-7901, A549, NCI-H1299 and L02 cells were cultured at 37 °C in 5% CO_2_ with RPMI-1640 supplemented with 10% FBS and 1% penicillin/streptomycin. Cells were passed every 2 days and restarted from frozen stocks upon reaching pass number 20.

### *In vitro* cytotoxic activity

*In vitro* cytotoxicity of the platinum compounds against human hepatocellular carcinoma (HepG-2), gastric carcinoma (SGC-7901), non-small-cell lung cancer (A549), and colorectal cancer (HCT-116) cell lines were measured by the MTT assays. Briefly, the cells were seeded in 96-well cultured plates at a density of 5000 cells/well. After overnight incubation (16 h), the cells were treated with the platinum complexes. After 48 h of incubation, 10 *μ*L of a freshly diluted 3-(4,5-dimethyl-2-thiazolyl)-2,5-diphenyl-2H-tetrazolium bromide (MTT) solution (5 mg/mL) were added to each well and the plates were incubated at 37 °C in a humidified 5% CO_2_ atmosphere for 4 h. At the end of the incubation period the medium was removed and the formazan product was dissolved in 150 μL of DMSO. The cell viability was evaluated by measurement of the absorbance at 490 nm, using an Absorbance Reader (BioRad). IC_50_ values (compound concentration that produces 50% of cell growth inhibition) were calculated from curves constructed by plotting cell survival inhibitory rate (%) versus drug concentration logarithm. All experiments were repeated in three times. The reading values were converted to the percentage of control (% cell survival). Cytotoxic effects were expressed as IC_50_ values.

### Reactions of complex 3 with DNA

Herring sperm DNA was dissolved in 10 mM phosphate buffer (pH 7.4) containing 10 mM NaClO_4_. The DNA concentration was determined by UV-vis spectra at 260 nm with an extinction coefficient of 6600 M^−1^. The mixture of DNA with platinum complexes were incubated at 37°C in the dark. The fluorescence was measured in 0.4 M NaCl to avoid the second fixation site of EtBr to DNA. EtBr (0.04 mg/mL) was added to the 0.01 mg/mL DNA solution before the fluorescence measurement. Fluorescence spectra were recorded under following conditions: scan speed 2000 nm min^−1^; excitation slit width was 5 nm and emission slit width was 10 nm. The excitation and emission wavelength was 530 nm and 592 nm, respectively.

### Cellular platinum uptake and DNA platination assay

The cellular uptakes of oxaliplatin, complex 3 and complex 3a were measured on A549, NCI-H1299 and L02 cells. The cells were seeded in 6-well plates overnight and then incubated with 10 μM drugs at 37°C in standard culture conditions. After 4 h, the cells were washed with PBS buffer (pH 7.4) for three times, and harvested by trypsinization. After re-suspension in PBS, the pellet was washed with PBS and collected per centrifugation (5910R, Eppendorf) at 500 g for 5 min at 4°C. The organelles were then isolated via differential centrifugation. All cellular compartments (mitochondria, lysosomes and nucleus) were isolated from A549 and NCI-H1299 cancer cells for direct comparative purposes. The supernatant phases discarded during the isolation of nuclei, lysosomes and mitochondria procedures were collected and formed the “residual” fraction. An aliquot of crude lysate after homogenization, nuclear, mitochondrial (pellet lysed via freeze and thaw cycles followed by 20 min incubation in ultrasonic bath), lysosomal and residual fraction was each used for protein quantification using the Bradford method [53]. The harvested cells were concentrated and digested by nitric acid for the ICP measurement. The cell numbers were counted before the digested. For the measurement of Pt concentration in cellular DNA in A549, NCI-H1299 cancer cells, DNA was isolated by applying Genomic DNA Mini Preparation Kit (Beyotime, China), and Pt content in DNA was analyzed by ICP.

### Apoptosis assessment

Annexin V-FITC apoptosis detection kit (Keygen, Jiangsu, China) assay was performed according to the manufacturer's protocol. Briefly, the A549 and NCI-H1299 cancer cells were treated with oxaliplatin, complex 3, complex 3a of 10 μM respectively for 24 h and washed with PBS. Then the cells were collected, resuspended in binding buffer (pH 7.5, 10 mM HEPES, 2.5 mM CaCl_2_ and 140 mM NaCl), incubated with Annexin V-FITC and then PI for 10 min in the dark at room temperature, the processed cells were analyzed by flow cytometry (FACSCalibur, Becton Dickinson) and a computer station running Cell-Quest software. (BD Biosciences, Franklin Lakes, NJ).

### Caspase-3 activation analysis

For the quantification of activation of caspase-3 in cancer cells, 5 × 10^5^ cancer cells were seeded in a 60 mm dish and allowed to adhere for 1 day. The cells were incubated with oxaliplatin, complex 3 and complex 3a at a concentration of 10 μM for 24 h to induce the activation of caspase-3. As a reference control, the cells were only incubated with fresh media at 37°C. Then, the cells were washed with PBS to eliminate the remaining drugs and harvested using trypsin-EDTA. All the groups of cells were collected into microtubes following incubation with 300 mL media containing 1 mL of FITC-DEVD-FMK (Caspase 3(active) FITC Staining Kit (ab65613)) for 1 h at 37°C under 5% CO_2_. Subsequently, centrifugation of the cells was performed to collect cell pellets and washed 2 times with washing buffer. Each group of cells was resuspended with 300 mL of the washing buffer and transferred 100 mL per well in a microtiter plate reader. The quantification of caspase-3 was evaluated as Relative Fluorescence Unit(RFU) using Fluorophotometer (Max Gemini EM, SoftMax® Pro 5, Molecular Devices Corp., Sunnyvale, CA) at an emission wavelength of 535 nm after excitation at 485 nm. The statistical significance was evaluated with one-way ANOVA test using Graph Pad Prism 4 software.

### Colony formation assay

Cells were trypsinized and plated at a density of 500 perplate. Fourteen days later, 50 μM oxaliplatin, complex 3 and complex 3a in cell culture medium were added to the plates, and cells were incubated for a further 7 days. Cells were then fixed with 3% paraformaldehyde, stained with crystal violet and imaged with a light microscope. The experiment was performed in triplicate. The number of colonies, defined as containing > 50 cells, was counted.

### Cell-cycle analysis

Cell cycle was analyzed by flow cytometry as described previously [54]. Data were analyzed with FlowJo software (TreeStar, Inc.).

### Mitochondrial transmembrane potential (ΔΨm) assessment

The electrical potential difference across inner mitochondrial membrane (ΔΨm) was monitored using the ΔΨm-specific fluorescent probe JC-1 (Molecular Probes Inc., Eugene, OR), a sensitive fluorescent dye. In brief, the A549 and NCI-H1299 cancer cells treated with 10 μM of oxaliplatin, complex 3 and complex 3a for 24 h were harvested with ice-cold PBS and resuspended in RPMI-1640 medium at a density of 0.5 ×10^6^ cells/ml, then the cells were permeabilized with 0.3% Triton X-100, washed with ice-cold PBS, incubated with 10 μM JC-1 for 15 min at 37°C in the dark and observed under a fluorescence microscope (Olympus IX51, Japan). Red fluorescence is attributable to a potential-dependent aggregation in the mitochondria. Green fluorescence, reflecting the monomeric form of JC-1, appeared in the cytosol after mitochondrial membrane depolarization. Relative fluorescence intensities were monitored using the flow cytometry (FACSCalibur, Becton Dickinson), and analyzed by the software Modfit and Cell Quest (BD Biosciences, Franklin Lakes, NJ) with settings of FL1 (green) at 530 nm and FL2 (red) at 585 nm.

### Measurement of ATP production

The ATP production assay kit (Haimen, Jiangsu, China) was used to measure intracellular ATP level according to the manufacturer's instructions. In brief, cells were treated with 10 μΜ oxaliplatin, complex 3 and complex 3a for 12 h, then incubated with 100 μL Nuclear Releasing Reagent for 5 min at 37°C with gentle shaking, followed by further incubation with 1 μL ATP monitoring enzyme. Detection was performed using the luminometer Orion II (Berthold DS, Bleichstr, Pforzheim, Germany).

### ROS measurement

The generation of ROS induced by complex 3 was determined with the cell permeant fluorogenic probe 2′,7′-dichlorodihydrofluorescein diacetate (H_2_DCFDA; Molecular Probes, Invitrogen, Darmstadt, Germany) as described [55]. H_2_DCFDA diffuses into the cell, where it is enzymatically converted by intracellular esterases and oxidized into the high fluorescence compound DCF, which allows the determination of H_2_O_2_, peroxynitrite anions and peroxyl radicals. Approximately 4×10^3^ A549 and NCI-H1299 cancer cells per well were plated on white bottom 96-well plates in extracellular fluid (140 nM NaCl, 3 mM KCl, 1 mM CaCl_2_, 1 mM MgCl_2_, 10 mM HEPES and 25 mM glucose; pH 7.4). After 24 h incubation, cells were treated with 10 μM oxaliplatin, complex 3 and complex 3a for 6 h at 37°C. Then, cells were loaded with 10 mM H_2_DCFDA for 30 min at 37°C and 5% CO_2_. Then, cells DCF generation was measured over time using a fluorometer (Fluostar BMG Labtech, Offenburg, Germany) at 492 nm excitation and 520 nm emission.

### H_2_O_2_ measurement

The production of H_2_O_2_ was determined with the Amplex^®^ Red reagent (10-acetyl-3,7-dihydroxyphenoxazine; Molecular Probes). In the presence of horseradish peroxidase (HPR), the Amplex^®^ Red reagent reacts in a 1:1 stoichiometry to produce the red-fluorescent oxidation product, resorufin. Consequently, resorufin generation allows the detection of the H_2_O_2_ released from biological compounds. Approximately 4 × 10^3^ A549 and NCI-H1299 cancer cells were plated on black bottom 96-well plates in 120 ml of working solution containing 40 mM Amplex^®^ Red reagent and 0.1 U/ml HPR, and incubated in the presence of 10 μM oxaliplatin, complex 3 and complex 3a for 2 h at 37°C. Resorufin generation was measured over time using a fluorometer (Fluostar BMG Labtech) at 544 nm excitation and 590 nm emission.

### Intracellular GSH measurement

A549 and NCI-H1299 cancer cells in 6 cm culture plates were treated as follows: 10 μM oxaliplatin, complex 3 and complex 3a. After 4 h, medium was removed and dishes were rinsed three times with cold PBS. The cells were harvested and cell numbers were counted. Then, intracellular GSH levels were measured using a GSH test kit (KeyGen KGT006). A549 and NCI-H1299 cancer cells without treatment were used as control groups.

### Gel electrophoresis

DNA cleavage produced by oxaliplatin, complex 3 and complex 3a was investigated by agarose gel electrophoresis. pET28a plasmid DNA (50 ng/mL) was used as the target. Appropriate dilutions of tested complexes were made, and the required volumes of solutions were added to achieve a set of concentrations in the range of 0–100 mm; pET22b DNA (5 mL, 0.20 mg) was added to each tube, and the mixtures of platinum complexes and pET22b plasmid DNA were then incubated at 37°C for 6 or 24 h. Afterward, the agarose gel (made up to 1% w/v) containing ethidium bromide was prepared with TA buffer (50 mm Tris-acetate, pH 7.5). The mixtures with loading buffer (1 mL) underwent electrophores is in agarose gel in TA buffer at 100 V for 60 min. Bands were imaged using a Molecular Imager (Bio-Rad, USA) under UV light.

### Comet assay

After the same treatment of oxaliplatin, complex 3 and complex 3a at 10 μM for 24 h, A549 cells (1×10^5^ cells) were combined with molten LMAgarose (Trevigen) at a ratio of 1:10 (v/v) and were immediately pipetted onto CometSlide (Trevigen). Slides were incubated at 4°C in the dark for 10 minutes, then immersed in prechilled Lysis Buffer and incubated at 4°C for 30 min. Slides were immersed in Alkaline Unwinding Solution, pH > 13 (200 mmol/L NaOH, 1 mmol/L EDTA) for 20 min at room temperature in the dark. Electrophoresis was done at 21 V for 30 minutes using Alkaline Electrophoresis solution (200 mmol/L NaOH, 1 mmol/L EDTA). The slides were washed twice in water for 5 minutes and once in 70% EtOH for 5 min, then dried overnight and visualized by microscopy. Under these conditions the formation of “comet tail” is indicative of SSBs, DBSs, and/or active excision repair of DNA cross-links.

### Analysis of SSB repair

SSB were detected by exposing cancer cells to 10 μM oxaliplatin, complex 3 and complex 3a, respectively, for 15 min, 30 min and 60 min in RPMI 1640 medium at 37°C. Then the cells were washed three times with PBSCMF (140 mM NaCl, 3 mM KCl, 8 mM Na_2_HPO_4_ and 1 mM KH_2_PO_4_). The numbers of SSBs were determined by an alkaline elution assay as previously described [56]. The numbers of SSB in untreated cancer cells were subtracted in all cases.

### Nuclear γH2AX foci analysis

For assessment of DSBs repair kinetics we detected the total nuclear volume for an average of 50–60 nuclei per time points and cell type. Briefly, A549 cells were grown to confluence in 35 mm petri-dishes with 0.17 mm glass bottom. After treatment of cisplatin, oxaliplatin and 3 at 50 μM for 8 h and 12 h, cells were fixed in 4% paraformaldehyde / PBS for 10 min at room temperature and incubated in nuclear extraction buffer (10 mM PIPES pH 6.8, 100 mM NaCl, 300 mM sucrose, 3 mM MgCl_2_, 1 mM EGTA, 0.5% Triton X-100) at 55 rpm on a rotating shaker for 15 min. Non-specific binding was blocked by incubation with 5% FCS/PBS/0.1% Triton X-100 for 1 h at RT. Immunostaining for MDC1 (1:400), and XRCC1 (1:1000) was followed by detection with species specific secondary AlexaFluor conjugates (1:1000; Invitrogen, Carlsbad, USA). Numbers of γH2AX foci were counted in 50–60 nuclei and plotted over time as previously described [57].

### Western blot analysis

After the treatment of the indicated concentration of 10 μM oxaliplatin, complex 3 and complex 3a for 24 h, A549 and NCI-H1299 cancer cells were collected and lysed in lysis buffer (100 mM Tris-Cl, pH 6.8, 4% (m/v) sodium dodecylsulfonate, 20% (v/v) glycerol, 200 mM β-mercaptoethanol, 1 mM phenylmethylsulfonyl fluoride, and 1 g/ml aprotinin). Lysates were centrifuged at 12,000 g for 0.5 h at 4°C. The concentrations of total proteins were measured using the BCA assay method with Varioskan spectrofluorometer and spectrophotometer (Thermo, Waltham, MA) at 562 nm. Protein (20–100 μg) prepared from the indicated cells was loaded per lane and electrophoresed in 8% or 10% sodium dodecyl sulfate polyacrylamide gel electrophoresis (SDS-PAGE), and then transferred onto polyvinylidene difluoride (PVDF) Immobilon-P membrane (Bio-Rad, USA) using a transblot apparatus (Bio-Rad, USA). The membranes were blocked with 5% (w/v) non-fat milk at 0.5 h at 37°C, followed by overnight incubation at 4°C with primary antibodies diluted in PBST. After washing with PBST, the membranes were incubated for 1 h with an IRDye´800 conjugated secondary antibody diluted 1:20000 in PBST, and the labeled proteins were detected with an Odyssey Scanning System (Li-COR., Lincoln, Nebraska, USA).

### Detection of acidic vesicular organelles (AVOs)

Flow cytometry with acridine orange staining as described in a previous study was used to detect and quantify the AVOs, one of the characteristicsof autophagy [58]. At the end of individual experiments, A549 cells were collected in phenol red-free RPMI 1640 medium. Green (FL-1) and red (FL-3) fluorescences of acridine orange were measured using a flow cytometer and CellQuest software (Becton Dickinson, San Jose, CA). The sum of the upper-left and upper-right quadrants of the cytogram was used to estimate the percentage of cells undergoing autophagy.

### Quantification of apoptotic and necrotic cells

The mode of cell death was analyzed by flow cytometry with annexin V/propidium iodide (PI) double-staining to detect membrane events according to a previous study [59]. After individual treatments, whole cells were collected in 4-(2-hydroxyethyl)-1-piperazineethanesulfonic acid (HEPES) buffer containing 10 mM HEPES (pH 7.4), 140 mM NaCl, and 2.5 mM CaCl_2_. Cells were subsequently stained with annexin V (2.5 μg/ml) and PI (2 ng/ml) for 20 min, followed by analysis by flow cytometry (Beckman Coulter). Cystograms of the four quadrants in the figure were used to distinguish normal (annexin V^−^/PI^−^), early apoptotic (annexin V^+^/PI^−^), late apoptotic(annexin V^+^/PI^+^), and necrotic cells (annexin V^−^/PI^+^). The sum of early apoptosis and late apoptosis is presented as total apoptosis.

### *In vivo* antitumor efficacy of complex 3

Thirty-five nude mice (BALB/c) with body weight range from 18–22 g, were supplied by Shanghai Laboratory Animal Center, China Academy of Sciences. Experimental protocols were in accordance with National Institutes of Health regulations and approved by the Institutional Animal Care and Use Committee. All animals were randomly divided into 5 groups. The A549 single-cell suspension in PBS (1×10^7^ cells per mouse) was injected subcutaneously into the right oxter of nude mice. When tumor grew to a size of 80–150 mm^3^ at 12 days, the mice were administrated via oxaliplatin (dosed intravenously at 5 mg/kg twice a week), complex 3 (dosed intravenously at 5 mg/kg once every three days), complex 3a (dosed intravenously at 5 mg/kg once every three days). The control group was administered glucose. The tumor growth was monitored by measuring the perpendicular diameter of the tumor using calipers every two days and calculated according to the formula:

Tumor volume (mm^3^) = 0.5 × length × width^2^

Growth curves were plotted using the average tumor volume within each experimental group at the set time points. The whole group of mice was sacrificed after the last treatment, then tumor weight was evaluated as the antitumor activity of the corresponding groups, and the kidneys, livers and tumors were excised for immunohistochemistry analysis. The body weight and physical state of the mice were measured simultaneously as an indicator of systemic toxicity.

### Blood stability test of complex 3 with rat plasma

To a volume of 0.6 mL rat plasma was added 10 μL of a DMSO solution of oxaliplatin and complex 3 (10 mM), resulting in a final concentration of 50 μM. At varying time points (0, 2, 4 and 8 h) at 37°C, a 300 μL aliquot was removed from the incubating blood. The ICP-MS measurement was served to confirm the blood stability of complex 3 in rat plasma.

### Pharmacokinetics studies

Twenty-four SD rats (240–250 g) were supplied by Shanghai Laboratory Animal Center, China Academy of Sciences. Experimental protocols were in accordance with National Institutes of Health regulations and approved by the Institutional Animal Care and Use Committee. SD rats composed of half male and half female were randomly divided into 2 groups: 1) oxaliplatin (5 mg/kg); 2) complex 3 (an equivalent dose to 5 mg oxaliplatin/kg). 0.6 ml of blood compounds were collected from orbital cavity at 5 min followed by 0.5, 1, 2, 4, 8, 12 and 24 h after intravenous administration, heparinized and then centrifuged at 4000 rpm for 20 min at 4°C. The supernatant plasma was collected for measurement of Pt content using ICP-MS. The apparent plasma half-life time was calculated using a Phoenix Win Nonlin 6.3 Program (Pharsight Cooperation, St. Louis Missouri, USA).

### Statistical analysis

All data in different experimental groups were expressed as mean ± SEM. These data shown in the study were obtained in at least three independent experiments. Statistical analyses were performed using an unpaired, two-tailed Student's *t*-test. All comparisons are made relative to untreated controls and significance of difference is indicated as *P < 0.05 and **P < 0.01.
